# HIV viral load as an independent risk factor for tuberculosis in South Africa: collaborative analysis of cohort studies

**DOI:** 10.7448/IAS.20.1.21327

**Published:** 2017-06-23

**Authors:** Lukas Fenner, Andrew Atkinson, Andrew Boulle, Matthew P. Fox, Hans Prozesky, Kathrin Zürcher, Marie Ballif, Hansjakob Furrer, Marcel Zwahlen, Mary-Ann Davies, Matthias Egger, for the International Epidemiology Database to Evaluate AIDS in Southern Africa (IeDEA-SA)

**Affiliations:** ^a^ Institute of Social and Preventive Medicine, University of Bern, Bern, Switzerland; ^b^ Department of Medical Parasitology and Infection Biology, Swiss Tropical and Public Health Institute, Basel, Switzerland; ^c^ University of Basel, Basel, Switzerland; ^d^ Department of Infectious Diseases, Bern University Hospital, University of Bern, Bern, Switzerland; ^e^ Khayelitsha ART Programme, Médecins Sans Frontières, Cape Town, South Africa; ^f^ Health Economics and Epidemiology Research Office, Department of Internal Medicine, School of Clinical Medicine, Faculty of Health Sciences, University of the Witwatersrand, Johannesburg, South Africa; ^g^ Departments of Epidemiology and Global Health, Boston University, Boston, MA, USA; ^h^ Division of Infectious Diseases, Department of Medicine, University of Stellenbosch and Tygerberg Hospital, Cape Town, South Africa; ^i^ Centre for Infectious Disease Epidemiology and Research (CIDER), School of Public Health and Family Medicine, University of Cape Town, Cape Town, South Africa

**Keywords:** tuberculosis, HIV, antiretroviral treatment, viral load, CD4 cell count, time-updated, incidence, opportunistic infection, prediction

## Abstract

**Introduction**: Chronic immune activation due to ongoing HIV replication may lead to impaired immune responses against opportunistic infections such as tuberculosis (TB). We studied the role of HIV replication as a risk factor for incident TB after starting antiretroviral therapy (ART).

**Methods**: We included all HIV-positive adult patients (≥16 years) in care between 2000 and 2014 at three ART programmes in South Africa. Patients with previous TB were excluded. Missing CD4 cell counts and HIV-RNA viral loads at ART start (baseline) and during follow-up were imputed. We used parametric survival models to assess TB incidence (pulmonary and extrapulmonary) by CD4 cell and HIV-RNA levels, and estimated the rate ratios for TB by including age, sex, baseline viral loads, CD4 cell counts, and WHO clinical stage in the model. We also used Poisson general additive regression models with time-updated CD4 and HIV-RNA values, adjusting for age and sex.

**Results**: We included 44,260 patients with a median follow-up time of 2.7 years (interquartile range [IQR] 1.0–5.0); 3,819 incident TB cases were recorded (8.6%). At baseline, the median age was 34 years (IQR 28–41); 30,675 patients (69.3%) were female. The median CD4 cell count was 156 cells/µL (IQR 79–229) and the median HIV-RNA viral load 58,000 copies/mL (IQR 6,000–240,000). Overall TB incidence was 26.2/1,000 person-years (95% confidence interval [CI] 25.3–27.0). Compared to the lowest viral load category (0–999 copies/mL), the adjusted rate ratio for TB was 1.41 (95% CI 1.15–1.75, *p* < 0.001) in the highest group (>10,000 copies/mL). Time-updated analyses for CD4/HIV-RNA confirmed the association of viral load with the risk for TB.

**Conclusions**: Our results indicate that ongoing HIV replication is an important risk factor for TB, regardless of CD4 cell counts, and underline the importance of early ART start and retention on ART.

## Introduction

In 2014, almost 1.5 million people died from tuberculosis (TB), and an estimated 9.6 million developed the disease worldwide [1]. TB is now the leading cause of death from an infectious disease, along with HIV/AIDS. HIV-positive patients are at high risk for opportunistic infections (OIs) such as TB. HIV infection and HIV-associated immunodeficiency are strong risk factors for TB, and in many low-income countries TB is the most common AIDS-defining illness [2–4]. Combination antiretroviral therapy (ART) has substantially improved the prognosis of HIV infection, and reduced the risk of OIs in both industrialized and low-income countries [5,6].

However, the risk of activation of latent TB and progression to clinical disease remains high in HIV-positive patients with high CD4 cells, possibly because HIV replication itself is associated with impaired protection against progression to active TB [7]. Indeed, patients with high levels of HIV replication appear to have a greater risk of OIs compared to patients on ART with suppressed HIV-replication and similar CD4 cell counts [8–10]. There is increasing evidence that ongoing HIV replication causes chronic immune activation, including the release of cytokines, and increased cell turnover, which in turn leads to a shift in lymphocyte phenotypes and to a reduced quality of the immune response [11].

Previous studies of the association of HIV-RNA viral load with TB incidence were conducted in high-income settings with a low TB incidence, and had a relatively small sample size. We analysed a large collaborative cohort of HIV-positive patients from three different ART programmes in South Africa to study the role of ongoing HIV replication as an independent risk factor for TB after starting ART.

## Methods

**Figure 1.**
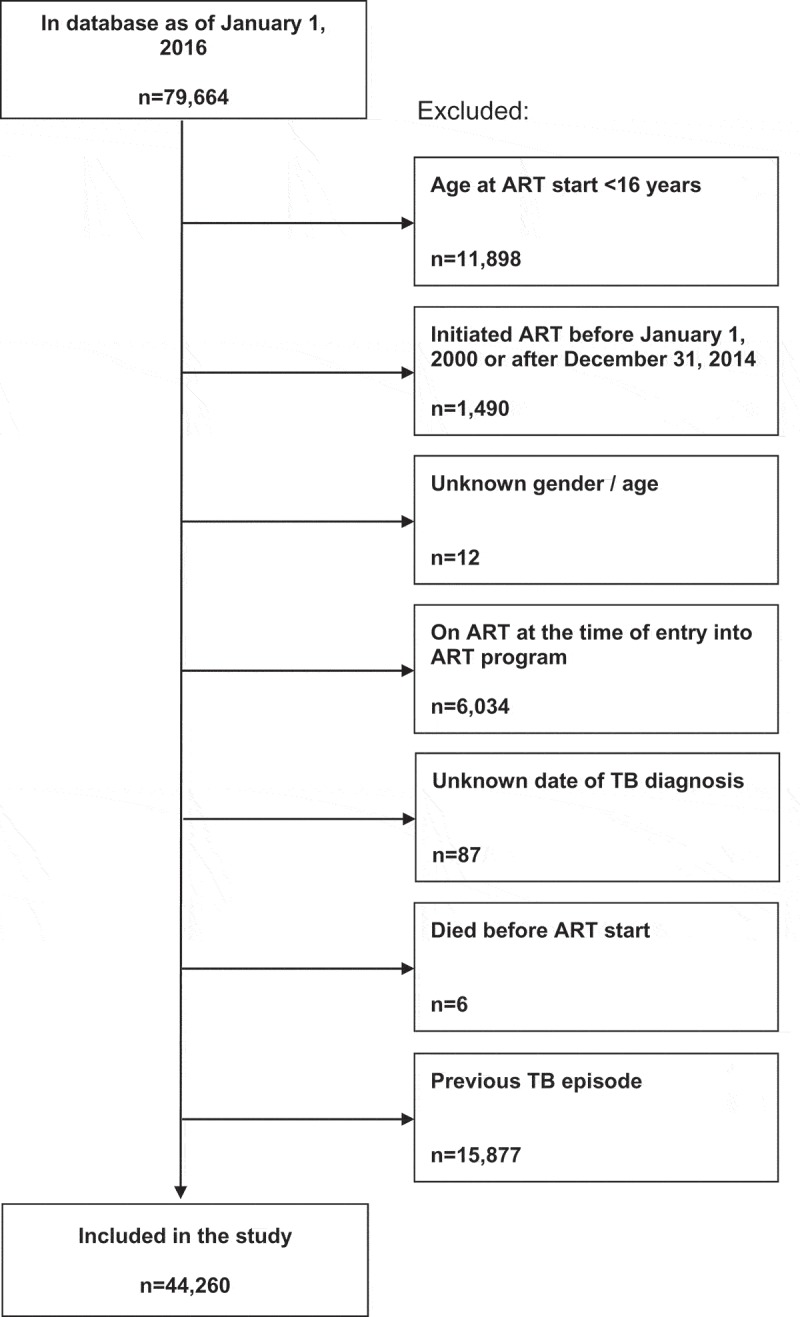
Selection of the study population. ART: antiretroviral treatment; TB: tuberculosis.

We included data from patients ≥16 years of age starting ART between 1 January 2000 and 31 December 2014 in three South African treatment programmes that participate in the International Epidemiology Databases to Evaluate AIDS in Southern Africa (IeDEA-SA, www.iedea-sa.org) [12], and also systematically record OIs and HIV viral load (Khayelitsha and Tygerberg in the Western Cape, Themba Lethu Clinic in Johannesburg). Data are collected as part of routine monitoring at enrolment and each follow-up visit, including TB symptoms. All study sites have local institutional review board or Ethics Committee approval to collect data and participate in IeDEA-SA. Children were excluded as they present a different study population, as well as patients with a previous TB episode. The selection of eligible patients is shown in Figure 1.

An incident TB episode was defined as a diagnosis and treatment start of TB (pulmonary or extrapulmonary) one month after ART initiation or later, as reported by each site [13,14]. Person-time was calculated from ART initiation (baseline) to TB diagnosis, death or last follow-up information. To account for missing values of CD4 cell count and HIV-RNA viral load at baseline and during follow-up, we generated 50 imputed datasets assuming missing at random using the MICE and Amelia II package in R [15]. The number of missing values are shown in Additional File 1. The imputation model included age, sex, CD4 count, viral load, observation time, and WHO clinical stage [16]. Results were combined with Rubin’s rules [17]. For the purpose of the imputation process, patients were expected to have laboratory tests every four months until the time of TB diagnosis, death or last follow-up information.

We used chi-squared tests for differences between groups in binary variables, and the Wilcoxon rank-sum test for continuous variables. We used parametric survival models to assess TB incidence stratified by levels of baseline CD4 cell count (0–99, 100–349, ≥350 cells/µL, unknown) and HIV-RNA (0–999, 1,000–9,999, ≥10,000 copies/mL, unknown). We estimated rate ratios adjusted for age, sex, baseline viral loads, CD4 cell counts, and WHO clinical stage, taking into account clustering by cohorts (ART programmes). Rate ratios were also calculated using a complete case dataset (without missing CD4 cell count and HIV viral load at start of ART). In addition, interactions between CD4 count and HIV-RNA on TB incidence were assessed by including interaction terms in the regression model. Finally, we used Poisson generalized additive regression models with time-updated CD4 cell counts and HIV-RNA values, smoothed by a regression spline for CD4 and HIV-RNA, and adjusting for age and sex.

All analyses were performed in Stata version 14.1 (Stata Corporation, College Station, TX, USA) and R 12.1 (R Development Core Team, Vienna, Austria).

## Results

We analysed 44,260 patients with a median follow-up time of 2.70 years (interquartile range [IQR] 0.99–5.02); 3,819 patients (8.6%) had an incident TB diagnosis one month after starting ART or later (median 1.05 years, IQR [0.33, 2.66]). Median age at ART initiation (baseline) was 34 years (IQR 28–41 years), and 30,675 (69.3%) patients were female. Baseline median CD4 cell count was 156 cells/µl (IQR 79–229) and the median HIV-RNA viral load was 58,000 copies/mL (IQR 6,000–240,000) (Table 1). Most patients were on an ART regimen based on two nucleoside reverse transcriptase inhibitors and one non-nucleoside reverse transcriptase inhibitor (41,104 – 92.9%); 3,156 (7.1%) were on other regimens. Patients who developed TB had lower baseline median CD4 cell counts (117 versus 161 cells/µl), higher baseline median HIV viral loads (111,000 versus 50,000 copies/mL), and more advanced disease (WHO clinical stage III/IV 22.2% versus 15.6%) compared to other patients, whereas distributions of age and sex were similar (Table 1).

**Table 1.** Baseline characteristics of patients in antiretroviral treatment (ART) programmes included in the study, overall and stratified by tuberculosis (TB) status

	All	Incident TB	No TB	
Characteristic	*n* = 44,260	*n* = 3,819	*n* = 40,441	*p-*Value
Age at start of ART, median (IQR), years	34 (28–41)	33 (28–40)	34 (29–41)	0.015
Female sex, *n* (%)	30,675 (69.4)	2,448 (64.1)	28,227 (69.8)	<0.001
Site of TB, *n* (%)	3,819	3,819	-	
Pulmonary	2,797 (73.2)	2,797 (73.2)	-	
Extrapulmonary	1,022 (26.8)	1,022 (26.8)	-	
CD4 cell count at ART start, median (IQR), cells/µl	156 (79–229)	117 (55–180)	161 (82–233)	<0.001
*No. of patients with value (%)*	36,773 (83.1)	3,227 (84.5)	33,546 (83.0)	
*Imputed values*	175 (96–270)	130 (67–205)	179 (100–277)	
HIV RNA viral load at ART start, median (IQR), copies/mL	58,000	111,000	50,000	<0.001
	(6,000–240,000)	(32,000–370,000)	(3,900–220,000)	
*No. of patients with value (%)*	7,205 (16.3)	924 (24.2)	6,281 (15.5)	
*Imputed values*	36, 013	107,102	27,472	
	(1,613–418,771)	(3,526–500,000)	(1,613–365,717)	
WHO clinical stage, *n* (%)				
*No. of patients with value (%)*	15,711 (35.5)	1,021 (26.7)	14,690 (36.3)	<0.001
I and II	13,187 (84.0)	794 (77.7)	12,393 (84.4)	
III	2,041 (13.0)	166 (16.3)	1,875 (12.7)	
IV	483 (3.0)	61 (5.9)	422 (2.9)	
*Imputed values for stage IV, %*	1,218 (2.8)	196 (5.1)	1020 (2.3)	
Treatment programme, *n* (%)	44,260			<0.001
Themba Lethu	15,711 (35.5)	1,021 (26.7)	22,412 (55.4)	
Khayelitsha	24,935 (56.3)	2,523 (66.1)	14,690 (36.3)	
Tygerberg	3,614 (8.2)	275 (7.2)	3,339 (8.3)	

IQR: interquartile range

**Figure 2.**
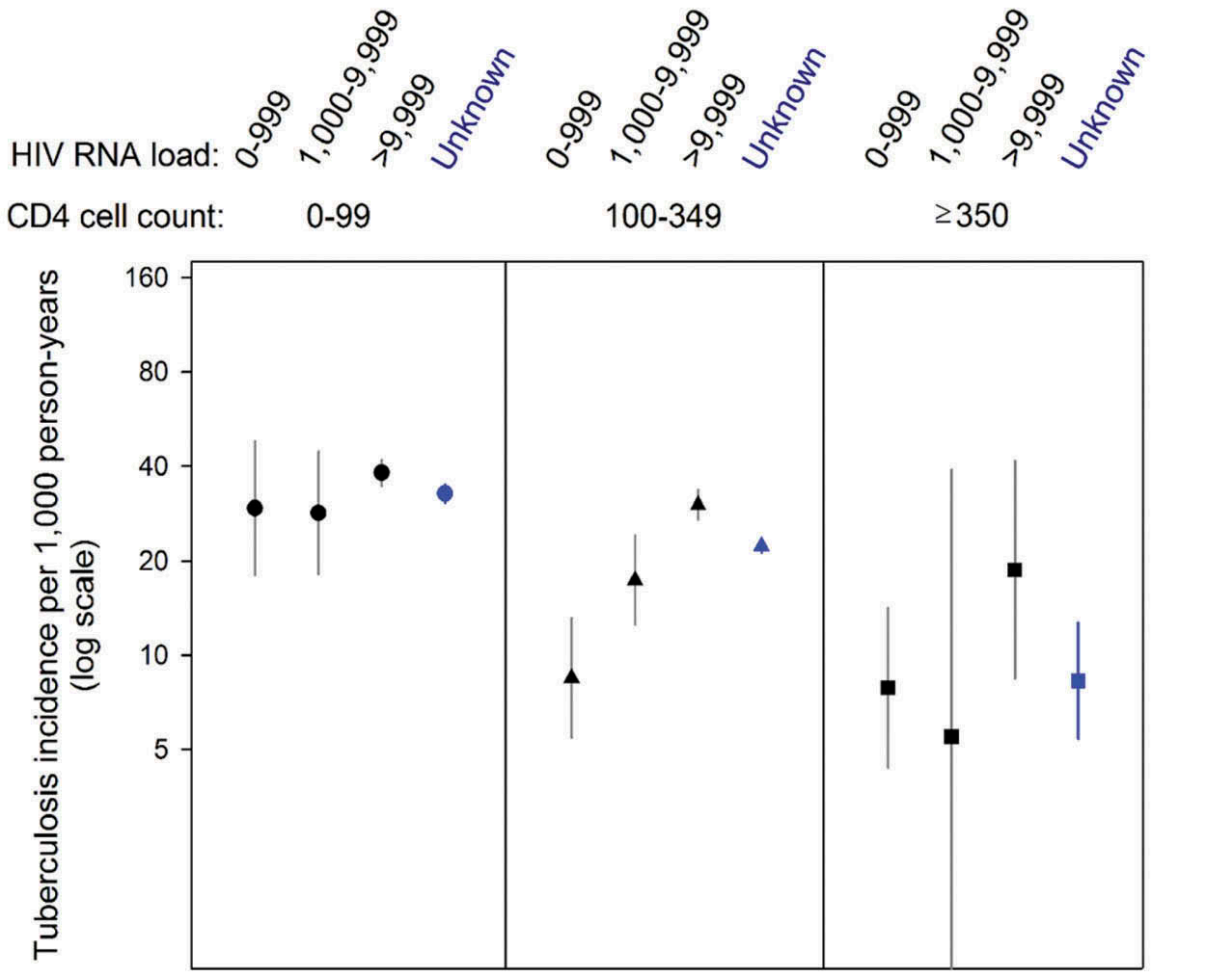
Crude incidence rates (per 1,000 person-years) of tuberculosis after starting antiretroviral therapy, by baseline levels of CD4 cell count (cells/μl) and HIV-RNA viral load (copies/mL). Based on 44,260 HIV-positive patients. Bars correspond to 95% confidence intervals. Unknown HIV-RNA viral load categories are shown in blue (unknown CD4 cell count category not shown).

During 146,008 person-years, TB incidence was 26.2 per 1,000 person-years (95% confidence interval [CI] 25.3–27.0). TB incidence was higher in patients with high HIV-RNA compared to patients with lower HIV-RNA, with this difference being almost fourfold in the intermediate and highest CD4 stratum (Figure 2). When comparing the highest with the lowest CD4 cell group (≥350 versus <100 cells/µl), the adjusted rate ratio for TB was 0.52 (95% CI 0.39–0.69, *p* < 0.001), as shown in Table 2. Comparing the highest with lowest HIV-RNA group (0–999 versus ≥10,000 copies/mL), the adjusted rate ratio for TB was 1.42 (95% CI 1.19–1.69, *p* < 0.001). There was no statistically significant interaction between the effects of CD4 count and HIV-RNA on TB incidence (*p*-value from test for interaction: 0.24). The complete case analysis showed similar results (Table 2).

**Table 2.** Adjusted rate ratios for tuberculosis (TB) according to CD4 cell count and HIV-RNA viral load at start of antiretroviral therapy (ART).

	All patients	Patients with incident TB	Imputed dataset analysis (*n* = 44,260)		Complete case dataset analysis (*n* = 6,707)	
Characteristic	*n* = 44,260	*n* = 3,819 (8.6%)	Adjusted rate ratio (95% CI)	*p*-Value	Adjusted rate ratio (95% CI)	*p*-Value
CD4 cell count at ART start, cells/µl, *n* (%)				0.001		<0.001
0–99	11,506	1,388 (12.1)	1		1	
100–349	23,116	1,800 (7.8)	0.78 (0.67–0.92)		0.85 (0.82–0.88)	
≥350	2,151	39 (1.8)	0.49 (0.36–0.66)		0.52 (0.30–0.93)	
HIV RNA viral load at ART start, copies/ml, *n* (%)				<0.001		<0.001
0–999	1,336	54 (4.0)	1		1	
1,000–9,999	710	57 (8.0)	1.23 (1.08–1.41)		1.43 (1.00–2.04)	
≥10,000	5,159	813 (15.8)	1.41 (1.17–1.71)		2.01 (1.35–2.99)	
Age at start of ART, years	-	-	0.99 (0.98–1.0)	0.02	0.99 (0.97–1.00)	0.12
Sex				<0.001		<0.001
Male	13,585	1,371 (10.1)	1		1	
Female	30,675	2,448 (8.0)	0.76 (0.70–0.82)		0.78 (0.70–0.87)	
WHO clinical stage, *n* (%)				<0.001		<0.001
I and II	27,553	1,693 (6.1)	1		1	
III and IV	15,664	2,060 (13.2)	1.84 (1.76–1.94)		1.98 (1.59–2.47)	

The complete case dataset included patients without missing CD4 cell count and HIV-RNA at start of ART. Model adjusted for age, sex, baseline viral loads, CD4 cell counts, and WHO clinical stage, taking into account clustering by cohorts (ART programmes). 95% CI: 95% confidence interval; IQR, interquartile range.

**Figure 3.**
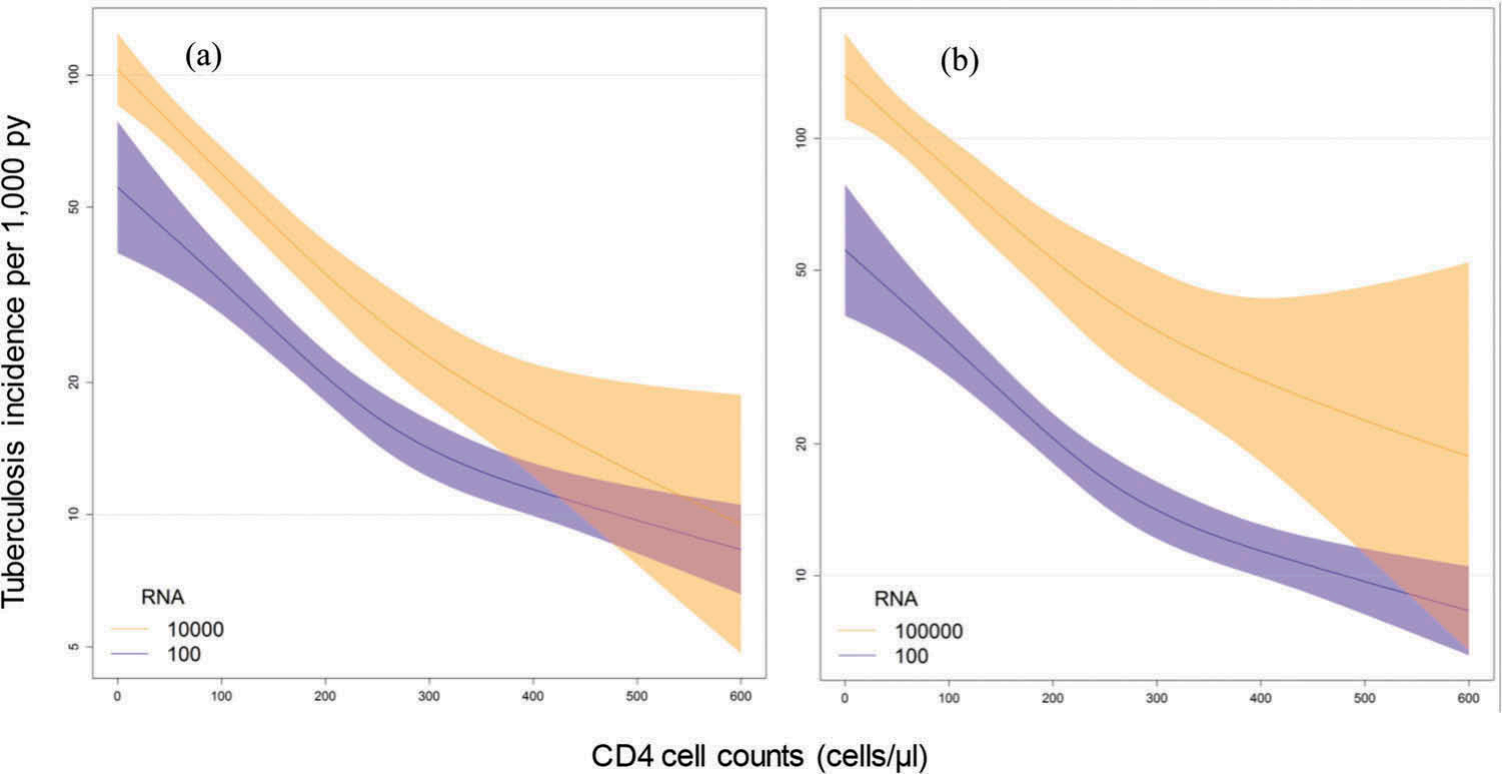
Influence of current CD4 cell count, and current HIV-RNA viral load on tuberculosis (TB) incidence. Models of TB incidence after starting antiretroviral therapy (ART) per 1,000 person-years, CD4 cell count (cells/µl), and HIV-RNA viral load (copies/mL) after imputation of missing CD4 cell counts and viral loads at start of ART and during follow-up, based on 44,260 HIV-positive patients. Curves represent patients with different HIV-RNA viral loads. (a) Viral load 100 vs. 10,000 copies/mL; (b) 100 vs. 100,000 copies/mL.

**Figure 4.**
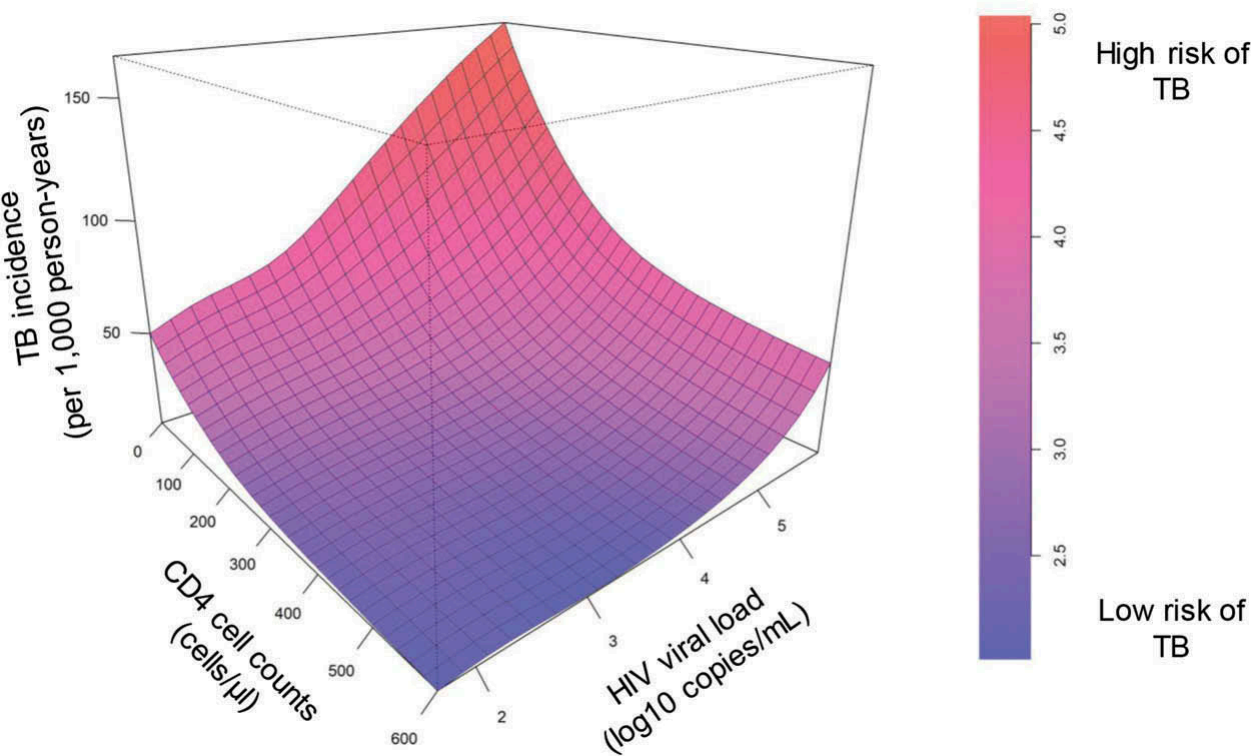
Three-dimensional model of current CD4 cell count (cells/µl), and HIV-RNA viral load (copies/mL) as predictors of tuberculosis (TB) incidence per 1,000 person-years after starting antiretroviral therapy (ART). Based on 44,260 HIV-positive patients after imputation of missing CD4 cell counts and HIV-RNA viral loads at start of ART and during follow-up. TB:  tuberculosis.

Time-updated analyses for CD4 cell count and HIV-RNA viral load fixed at different HIV-RNA levels confirmed the effect of viral load on the risk of TB (Figure 3), although CIs were wide in the range of higher CD4 cell counts. Similar results were obtained in a complete case analysis (Additional file 2, Figure). Figure 4 shows a three-dimensional model of CD4 cell count and HIV-RNA to predict TB incidence after starting ART.

## Discussion

We analysed HIV-positive patients starting ART in three large HIV treatment programmes in South Africa. We found that HIV-positive TB patients with ongoing HIV replication as determined by plasma HIV viral loads are at increased risk for TB.

Viral replication is an independent risk factor for TB during ART, regardless of CD4 cell counts. This is supported by both the analysis of TB incidence at baseline, as well as the analysis of time-updated CD4 cell counts and HIV-RNA viral loads on a continuous scale. CD4 cell counts in peripheral blood are a useful marker of immune competence in HIV-positive patients, but ongoing HIV replication as measured by the plasma HIV-RNA viral load has previously been suggested as a CD4 cell count independent risk factor for OIs in patients on ART [7–9]. Even though ART reduces the risk of TB substantially, the risk remains high in HIV-positive people, even in countries with low TB transmission [18,19].

Our findings show that HIV replication is a useful tool to predict the risk for TB after starting ART. This supports the current WHO guidelines to scale-up viral load testing in low-income countries to monitor adherence to ART and treatment failure. The recent results from clinical trials and the newly issued WHO guidelines state that ART should be initiated in all people diagnosed with HIV, regardless of CD4 cell counts at time of diagnosis [20]. However, many obstacles will need to be overcome before the new guidelines will be implemented widely in sub-Saharan Africa which carries the highest HIV-TB burden [21]. Until recently, ART was not generally recommended in HIV-positive patients with CD4 cell counts above 500 cells/µL. However, the risk of activating latent TB infection and fast progression to disease remains high in the group of HIV-positive patients with higher CD4 cells, particularly in patients with high HIV-RNA viral loads as shown here. This could possibly be explained because HIV replication itself is associated with a distorted immune system and impaired protection against progression to disease [11]. These mechanisms include increased cell turnover, activation, differentiation, and cytokine release [11]. This is demonstrated by the fact that the immune response to vaccines such as yellow fever is reduced in patients with ongoing HIV replication [22].

HIV-positive patients with high HIV viral loads are at high risk of TB, regardless of CD4 cell counts, and may therefore particularly benefit from administration of isoniazid preventive therapy (IPT) to reduce the risk of developing TB. Therefore, HIV viral load monitoring in patients on ART can be an important tool to identify patients at highest risk of TB who would benefit most from long-term IPT. In high TB incidence regions, the scale-up of IPT to prevent progression from infection to TB disease is still challenging as shown in a survey among ART programmes of the IeDEA collaboration [23]. In addition, randomized clinical trials showed a limited efficacy of IPT, and the benefit of IPT might be only short-term in high TB prevalence settings with ongoing TB transmission and a high risk of re-exposure [24–26]. Individuals with proven TB infection (e.g., tuberculin skin test positive) appear to benefit most from IPT [26], but reliable testing for latent TB is difficult to perform in low-income settings. However, IPT could be an additional tool that complements early start of ART to further reduce the risk of TB [27,28].

A limitation of our study was the missing values for HIV-RNA which is not routinely performed at start of ART in South Africa. We addressed this by imputing HIV-RNA values at baseline and during follow-up. Another limitation is potential residual confounding by factors not captured in the dataset, and the potential under-ascertainment of TB. However, we restricted our study to cohorts that systematically collect information on OIs. Another limitation was the heterogeneity between the included ART programmes [13]. To address this, we also calculated risk ratios, adjusted for the most important confounding factors.

## Conclusions

We found that ongoing HIV replication is an important risk factor for TB, regardless of CD4 cell counts. Furthermore, viral load values in addition to CD4 cell counts should be used to predict the risk of TB after starting ART and during follow-up. Our study underlines the importance of early start of ART in HIV-positive persons with a high viral load and continuous retention on effective ART. Further studies are needed during the ongoing scale-up phase of universal HIV-RNA viral load monitoring to document its usefulness to predict the risk of TB after starting ART and the effectiveness of IPT.

## Notes

To access the supplementary material to this article please see Supplemental Files under Article Tools online.
